# Applying genomic approaches to delineate conservation strategies using the freshwater mussel *Margaritifera margaritifera* in the Iberian Peninsula as a model

**DOI:** 10.1038/s41598-022-20947-5

**Published:** 2022-10-07

**Authors:** S. Perea, S. L. Mendes, C. Sousa-Santos, P. Ondina, R. Amaro, J. Castro, E. San-Miguel, C. S. Lima, M. Garcia, V. Velasquez, P. Garcia-Roves, D. Fernández, R. Araujo, V. C. Sousa, J. Reis

**Affiliations:** 1 MARE-Marine and Environmental Sciences Centre/ARNET-Aquatic Research Network, Faculdade de Ciências da Universidade de Lisboa, Campo Grande, 1749-016 Lisbon, Portugal; 2 Instituto de Biología, Departamento de Zoología, Universidad Nacional Autónoma de México, Tercer Circuito Exterior S/N, C.P. 04510 Mexico City, Mexico; 3 cE3c-Centre for Ecology, Evolution and Environmental Changes, Department of Animal Biology, Faculdade de Ciências da Universidade de Lisboa, Campo Grande, 1749-016 Lisbon, Portugal; 4 MARE-Marine and Environmental Sciences Centre/ARNET-Aquatic Research Network, ISPA-Instituto Superior de Ciências Psicológicas, Sociais e da Vida, Rua Jardim do Tabaco, 34, 1149-041 Lisbon, Portugal; 5 Departamento de Zooloxía, Xenética e Antropoloxía Física, Universidade de Santiago de Compostela, Campus Terra, 27002 Lugo, Spain; 6 IBADER-Instituto de Biodiversidade Agraria E Desenvolvemento Rural, Campus, Universidade de Santiago de Compostela, Campus Terra, 27002 Lugo, Spain; 7 Dirección General del Medio Natural y Desarrollo Rural, Oviedo, Principado de Asturias Spain; 8 Biosfera-Consultoría Medioambiental, C/Candamo no. 5,, C.P. 33012 Oviedo, Asturias Spain; 9 Museo Nacional de Ciencias Naturales - CSIC, C/José Gutierrez Abascal, 2, 28006 Madrid, Spain

**Keywords:** Ecology, Evolution, Genetics, Ecology, Environmental sciences

## Abstract

Effective conservation actions to counteract the current decline of populations and species require a deep knowledge on their genetic structure. We used Single Nucleotide Polymorphisms (SNPs) to infer the population structure of the highly threatened freshwater pearl mussel *Margaritifera margaritifera* in the Iberian Peninsula. A total of 130 individuals were collected from 26 locations belonging to 16 basins. We obtained 31,692 SNPs through Genotyping by Sequencing (GBS) and used this dataset to infer population structure. Genetic diversity given as observed heterozygosity was low. Pairwise F_ST_ comparisons revealed low levels of genetic differentiation among geographically close populations. Up to 3 major genetic lineages were determined: Atlantic, Cantabrian and Douro. This structure suggests a close co-evolutionary process with brown trout (*Salmo trutta*), the primordial fish host of this mussel in the studied area. Some sub-basins showed some genetic structuring, whereas in others no intrapopulation differentiation was found. Our results confirm that genetic conservation units do not match individual basins, and that knowledge about the genetic structure is necessary before planning recovery plans that may involve relocation or restocking. The same reasoning should be applied to strictly freshwater species that are sessile or have restricted dispersal abilities and are currently imperiled worldwide.

## Introduction

The understanding of genetic structure and diversity patterns of highly endangered organisms is crucial to guarantee the implementation of effective conservation actions that counteract the current decline of species and populations, ensuring their long-term preservation. The application of Single Nucleotide Polymorphisms (SNPs) to address evolutionary and conservation issues has become widely extended in non-model organisms^1,2^. Indeed, in the last fifteen years, the use of SNPs in population genetics has been developed as an extensively used genomic tool to assess patterns of genetic variation in populations. Advantages of SNPs include the higher number of analyzed loci (thousands or hundreds of thousands), which results in a more extensive screening of the genome, a lower genotyping error, a higher reproducibility among laboratories and more precise estimates of diversity due to the high mutation rate of microsatellites, which may lead to underestimate heterozygosity^3^. However, challenges in the use of SNPs still remain, such as the requirement of a large number of markers to obtain robust estimations of genetic variation among populations, when compared to multi-allelic microsatellites that, as an advantage over SNPs, provide higher levels of polymorphism, a faster evolutionary rate and low levels of ascertainment bias^4^. Consequently, although the use of SNPs has become increasingly more popular, microsatellites should not be disregarded. In fact, both kind of markers may be considered as complementary in Evolutionary Biology, as both may respond to different issues. For example, while adaptive processes cannot be assessed by the use of neutral markers such as microsatellites (located in non-coding regions), some SNPs (located in coding and non-coding regions) may be under selection, providing information about demographic (drift) and functional (selection) processes. However, microsatellites may perform more efficiently in relatedness and parentage analyses as a consequence of being multi-allelic markers (^5^ and references therein). Although SNPs and microsatellites may be equally valid and comparable in the analysis of the neutral genetic variation, the one analyzed in this paper, some studies comparing SNPs and microsatellites-based approaches with the same sampling set have advocated for the use of SNPs to attain more reliable inferences of patterns of genetic structure and diversity in different aquatic organisms, such as the brown trout^6^. Therefore, the analysis of SNPs, the markers used in this study, may be considered an efficient genomic tool to investigate evolutionary patterns and to make grounded decisions regarding conservation priorities for the most threatened fauna (e.g. Iberian lynx:^7^).

**Figure 1:**
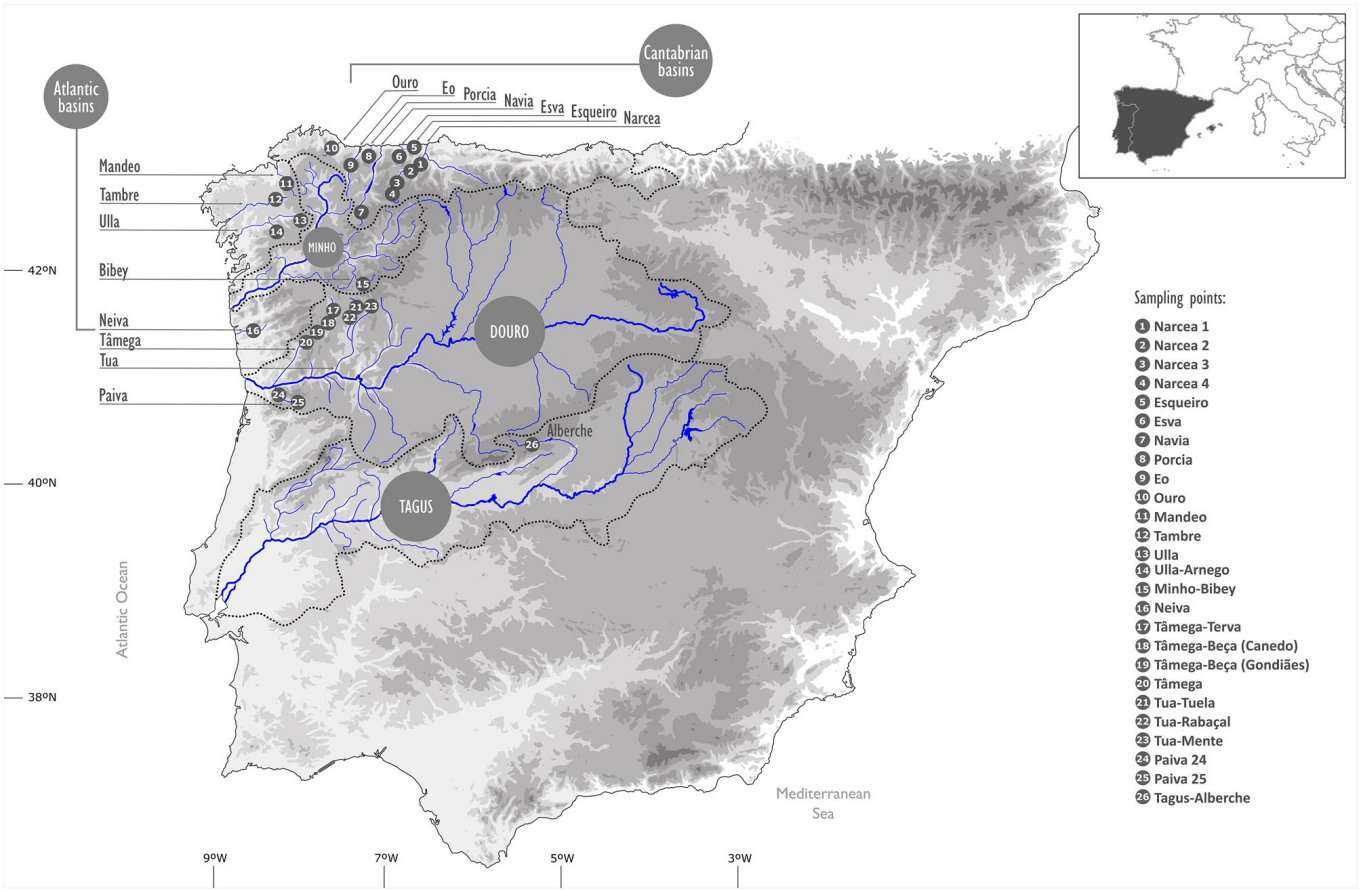
Sample locations for DNA collection.

Freshwater mussels from the order Unionida are worldwide distributed and cited amongst the most endangered animals in the world, because of the continuously increasing human activity that impacts the aquatic environment, which led to severe habitat reduction and strong population decline, combined with the unique biological traits of these organisms^8,9^. The freshwater pearl mussel *Margaritifera margaritifera* L. is a threatened species listed as critically endangered in Europe and included in the European Habitats Directive under Annexes II and V, and in Appendix III of the Bern Convention^9^. It has an Holarctic distribution, with populations in rivers on both sides of the Atlantic Ocean, ranging from the USA and Canada and from Russia and Scandinavian countries to the Iberian Peninsula^9^, that constitutes the southern limit of its distribution range. Here it is mainly located along several hydrological basins from the northwestern quadrant, from Asturias in the Cantabrian coast to the Douro river basin in the southern edge, and one isolated population in the Tagus Basin^10^ (Fig. 1).

The life cycle of *M. margaritifera* is complex. It can reach a lifespan of 100 years or more^8^, although Iberian specimens do not usually reach this age^10^. Its life cycle includes an obligatory parasitic larval stage. The larvae, called glochidia, need to infect salmonid fish, with the Atlantic salmon (*Salmo salar*) and the brown trout (*Salmo trutta*) being the preferred hosts^8^. Some of the healthiest *M. margaritifera* populations are located in rivers with massive salmon populations, but in most areas where the species is distributed, salmon populations are extinct or near extinction, and glochidia infect *Salmo trutta* populations more frequently or even exclusively^11^. In this sense, a co-evolutionary relationship between freshwater mussels and their fish host (*S. salar* and *S. trutta* for *M. margaritifera*) has been established, although some authors consider it to be more a symbiosis-commensalism than a parasitism relationship^12^.

The conservation status of *M. margaritifera* is poor. Indeed, during the last century, its European populations have suffered an estimated decline of around 90% because of habitat loss and fragmentation, overexploitation for pearl extraction, pollution and the introduction of invasive species^8,13^. The decline of host fish (salmonids) in most of the rivers inhabited by the freshwater pearl mussel, especially in southern European regions^14,15^, could also have contributed to the decline of *M. margaritifera* populations and must be considered in the future trend of the species^16^. In the Iberian Peninsula, populations of this species have an additional constraint, as southern regions might be more susceptible to climatic change associated to a stronger decrease in precipitation and an increase of more unstable hydrological conditions than in northern latitudes, which has been reported as a potential extinction risk for *M. margaritifera*^16^.

Because of the continuous threats to populations, several conservation actions have been taken in the last decades that aim to reduce or revert the species decline. These include habitat protection and restoration, host fish restocking, specimens’ relocation and captive breeding for restocking or reintroductions^17,18^. Relocation, restocking, and reintroduction actions pose questions about the genetic compatibility between populations as well as genetic-based adaptations to local environments that have been poorly addressed^18^, besides ethical issues regarding the anthropogenically-mediated mixture of distinct evolutionary lineages. Thus, the long-term preservation of this highly endangered species can greatly benefit from genomic studies^19^, since the analysis of SNPs can act as a powerful tool for describing the genomic variation of the freshwater pearl mussel at a wider genome scale, which is imperative for the effective implementation of conservation actions as those described above.

Several population-scale studies have tried to understand the genetic structure and diversity of *M. margaritifera* in the Iberian Peninsula, most of them focusing on populations from the Spanish northwestern region of Galicia^20,21^. Nevertheless, the whole distribution range of Iberian *M. margaritifera* has not been analyzed to date. These previous studies based on microsatellite markers have shown populations with a high degree of structuring and reduced genetic diversity^20^. High degrees of population structure have also been identified in central and northern European populations of freshwater pearl mussels, but in this case the degree of genetic diversity was variable, often with higher values^22,23^. Contrary to these genetic patterns, North American populations of *M. margaritifera* are characterized by high levels of genetic diversity and low degree of genetic differentiation, with a probable single panmictic population, and its conservation status has been considered as relatively safe in comparison with European populations^24,25^. Studies based on SNP markers for Iberian *M. margaritifera* populations have not been performed yet. Only two SNP-based studies for *Margaritifera* were performed in a restricted area of North America (*M. margaritifera*:^24^; *M. hembeli*:^26^), as well as one study in the unionid genus *Cyprogenia*, endemic to North America^27^.

The aims of this study were (1) to identify the evolutionary units of *M. margaritifera* in the Iberian Peninsula through the analysis of its genetic structure based on SNPs; (2) to estimate the genetic diversity of these evolutionary units; (3) to determine the factors that better explain their genetic structure and diversity; (4) to define conservation units for conservation purposes; and finally, (5) to propose how the information on genetic patterns and evolutionary units should be incorporated in conservation and management plans.

## Material and methods

### Sampling

A total of 130 individuals of *M. margaritifera* were collected from 26 locations at 16 hydrological units, corresponding to coastal Cantabrian and Atlantic basins and sub-basins belonging to the large Douro watershed (Fig. 1; Table S1 in Supporting Information). Specimens were visually located in rivers with previously known populations, using glass-bottom buckets or by snorkeling or scuba-diving. For genomic analyses, a small piece of the foot muscle from each individual was collected in situ and preserved in ethanol 95% until DNA extraction. For some populations (rivers Alberche, Mandeo, Ouro and Tambre) previously collected tissues preserved at -80^◦^C at the Museo Nacional de Ciencias Naturales in Madrid were used (Table S1 in Supporting Information).

### DNA extraction and genotyping by sequencing

Total genomic DNA was extracted from preserved tissue for each individual using the commercial kit DNeasy Blood and Tissue (QIAGEN), following the manufacturer’s instructions. For some samples, DNA concentration rendered too low for library construction and an ethanol precipitation protocol was used to increase it. The final concentration of all samples was determined using the Qubit® 2.0 Fluorometer and DNA fragmentation was verified in a 1% agarose gel. Samples were subjected to a paired-end Genotyping by Sequencing (GBS) protocol (adapted from Elshire et al., 2011), which was performed at LGC Genomics, GmbH, Germany. DNA was fragmented using the restriction enzyme MslI, libraries were constructed and sequenced using Illumina NextSeq with a read length of 150 base pairs (bp).

### Data processing and genotype calling

Sequence quality of reads was assessed using FastQC 0.11^28^ and MultiQC^29^ and all data was included in the subsequent analyses. The *process_radtags* program of STACKS v.2.5^30^ was used to truncate all reads to the same length (135 bp) and to discard reads with low quality scores and uncalled bases, using the default settings for the window size (0.15 of the read length) and the base quality threshold (10 Phred score). The Stacks software only cuts the final end of the reads, therefore, Trimmomatic v.0.36^31^ was used to eliminate the first 5 bases in all sequences. The final length of reads was 130 bp. All sequences passed quality standards to be included in the subsequent analyses after process_radtags analysis (Phred score > 30).

The de novo pipeline from the STACKS v.2.5 software^30^ was used to build a catalog of loci. A subset of individuals covering all sampled sub-basins was used in the construction of the catalog; these individuals are represented in Table S1. In the de novo Stacks pipeline, first the identical reads are grouped in stacks and these stacks are merged into loci in each sample individual. Then, a catalog is created by determining which loci are homologous across all the analyzed samples. Different m (minimum number of reads required to form a stack), M (number of mismatches allowed between loci when processing a single individual) and n (number of mismatches allowed between loci during the construction of the catalog) parameters were tested to select the optimal ones in the construction of the catalog, following the recommendation of^31^. The program *populations* included in the software STACKS v.2.5^30^ was used to test the number of genotyped loci and variant sites. For the subset of individuals used for the catalog, the parameters that maximized the number of SNPs were M = 1, m = 4 and n = 2, and the resulting catalog was used in downstream analyses.

Given the possibility that forward and reverse sequences of the same DNA fragment were treated as different loci, similar reads within the catalog were clustered using CD-HIT-EST from the CD-HIT v. 4.8.1 software^32^ with a word length of 10 and a sequence identity threshold of 0.98. The paired pre-processed sequences (those truncated to 130 bp) of all individuals were aligned to this catalog using BWA-MEM from bwa v. 0.7.17^33^ with default parameters. We sorted the output alignments and removed unmapped reads using Samtools v1.10^34^. We used the software Freebayes v.1.3.1^35^ to carry out the SNP-calling based on haplotypes for variant detection and considering the cleaned catalog as reference to genotype SNPs, discarding reads and bases with low quality, and without using Hardy–Weinberg equilibrium priors (-p 2-min-mapping quality 30-min-base-quality 20-hwe-priors-off). The output from Freebayes (.vcf file) was subjected to different filters in order to (1) eliminate indels; (2) remove genotypes with a depth of coverage (DP) lower than 4; (3) remove sites with excess of heterozygosity when pooling all individuals; (4) remove sites with more than 60% missing data and, finally, (5) eliminate a minimum allele frequency below 1%. These filters were applied using a combination of options from VCFtools v0.1.15^36^ and BCFtools v1.6^34^. The final dataset comprised 31,692 SNPs with 32.79% missing data and 119 individuals from the initial 130.

### Genetic diversity and genetic structure analyses

Genetic diversity was evaluated by analyzing the expected and observed heterozygosity at different hierarchical levels (sampling location and hydrological units) using custom scripts. The program *populations* of STACKS v. 2.54^30^ was used to estimate genetic diversity statistics for each population, using the final vcf file (31,692 filtered SNPs): number of private alleles, number of polymorphic sites, nucleotide diversity, and F_IS_ values (inbreeding coefficient). Significance for F_IS_ values was estimated in Arlequin v.3.5 using 10,000 permutations tests^37^.

Genetic structure was inferred by calculating pairwise F_ST_ values for all population pairs using the Weir and Cockerham's estimator^38^ in the Arlequin v. 3.5 software^37^. The corresponding significance (p-values) were computed with 10,000 permutations and were adjusted for multiple comparisons by using Bonferroni (Rise, 1989) corrections. To investigate population structure at different hierarchical levels we performed an Analysis of Molecular Variance (AMOVA)^37^. More specifically, we tested three distinct grouping schemes for which, in decrescent order of stratification, we considered as "populations" in the context of AMOVA analyses: (1) within individualized sampling locations considered as independent (without any grouping in higher hierarchical geographical levels); (2) among sampling locations grouped within individualized hydrological sub-basins; and (3) among individualized sub-basins (individuals grouped by sub-basin, without taking in consideration different sampling locations within sub-basins). Significance was tested with 10,000 permutations.

Finally, to investigate population structure using individual-based methods, we determined the ancestry proportions from specified number of clusters (K) using sNMF v.2.0 from the R package LEA^39^ and ADMIXTURE v1.3^40^, testing values of K from 1 to 16 (number of sub-basins) and performing 100 independent runs for each value of K. For sNMF, we identified the most plausible K value as the one with the lowest cross-entropy. For ADMIXTURE, we identified the best K value as the one with the lowest fivefold cross-validation error and for 2 ≤ K ≤ 3, we assessed similarity across the 100 replicates using the Greedy algorithm implemented in CLUMPP v1.1.2^41^.

### Association between genetic and geographic distances

For river basins having more than two sampling locations (Douro, Narcea and Ulla), approximate geographic distances between sampling locations were measured by hand (in km), following the natural meandering of rivers, using the Path tool available in Google Earth Pro version 7.3.3.7786 (© Google LLC). Insurmountable barriers for fish (dams, weirs and waterfalls) were mapped along with the drawing of pathways.

To evaluate the possible existence of isolation by distance, linear regression analyses were conducted using approximate geographic distances between sampling locations (log transformed) and their respective pairwise F_ST_/(1-F_ST_) estimates (using pairwise F_ST_ values at location level—Table S3).

## Results

### Genetic diversity and genetic structure

Genetic diversity at the individual level, measured as observed heterozygosity, did not exhibit a geographic pattern and values were generally low for most populations. Values of average observed heterozygosity across all SNPs ranged from 0.045 to 0.066 (Table 1). The highest values were found in Eo (Cantabrian basin) and Neiva (Atlantic basin) along with three different tributaries of Tua sub-basin of the Douro river basin (Rabaçal, Mente and Tuela) (see Fig. 1 for river locations). The lowest values of observed heterozygosity (< 0.050) were those from the Narcea and Porcia Cantabrian basins, and from the Mandeo and Bibey Atlantic basins. This agrees with measures of genetic diversity at the population level, measured as expected heterozygosity (Table 1): Bibey (Atlantic lineage) exhibited the lowest value (0.043) and one Narcea population (0.045) whereas the highest one (0.059) were found in Eo (Cantabrian basin) and Tuela (Douro basin). Eo and Neiva populations also showed the highest values of nucleotide diversity, which ranged from 0.057 to 0.059, whilst the lower nucleotide diversity values (≤ 0.045) were found in two localities of the Narcea river and in the Mandeo basin (Table 1). In general, the locations belonging to the large Douro basin showed a smaller number of private sites (25–79) than the remaining basins (76–225, excluding the 35 of Porcia, Table 1), suggesting a higher connectivity. The highest values of private sites were found in the Atlantic Ulla basin (225) and in Alberche (167) river (Tagus basin) (Table 1), indicating that these are probably more isolated than the other sampled locations. Outside the Douro hydrological basin, the four locations in the Narcea basin showed a smaller number of private sites than the rest of non-Douro basins (Table 1). The F_IS_ values, either positive or negative, were always close to zero for all populations and non-significant for all of the locations (Table S2).

**Table 1:** Genetic diversity statistics: expected (H_exp) and observed (H_obs) heterozygosity, nucleotide diversity (π) and private sites for the dataset of 26 locations and 16 hydrological basins.

Region	Population	H_exp	H_obs	π	Private sites
Cantabrian	Narcea (all)	0.050	0.048	–	–
	Narcea 1	0.049	0.048	0.049	104
	Narcea 2	0.052	0.050	0.054	76
	Narcea 3	0.045	0.045	0.045	81
	Narcea 4	0.047	0.047	0.049	77
	Esqueiro	0.049	0.053	0.049	105
	Esva	0.047	0.050	0.047	122
	Porcia	0.047	0.049	0.051	35
	Navia	0.053	0.057	0.054	91
	Eo	0.059	0.060	0.059	133
	Ouro	0.056	0.058	0.057	114
Atlantic	Mandeo	0.046	0.047	0.046	114
	Tambre	0.055	0.056	0.055	162
	Ulla (all)	0.057	0.058	–	–
	Ulla	0.052	0.053	0.052	225
	Arnego	0.054	0.059	0.055	144
	Bibey	0.043	0.045	0.043	114
	Neiva	0.057	0.062	0.057	97
Douro	Douro (all)	0.056	0.057	–	–
	Tâmega (all)	0.052	0.058	–	–
	Tâmega	0.051	0.057	0.052	40
	Beça Gondiães	0.049	0.055	0.049	52
	Beça Canedo	0.051	0.056	0.053	25
	Terva	0.054	0.061	0.055	52
	Tua (all)	0.058	0.063	–	–
	Mente	0.058	0.063	0.058	58
	Rabaçal	0.052	0.060	0.053	50
	Tuela	0.059	0.066	0.059	75
	Paiva (all)	0.048	0.051	–	–
	Paiva 25	0.046	0.051	0.048	79
	Paiva 24	0.048	0.051	0.047	56
Tagus	Alberche	0.048	0.051	0.051	167

We found a clear genetic population structuring within the Iberian Peninsula. Pairwise F_ST_ comparisons based on the Weir and Cockerham’s estimator revealed low levels of genetic differentiation between geographically close basins, such as Navia—Esqueiro Cantabrian basins with F_ST_ close to zero (Table 2), or between locations within the same basin/sub-basin, as the four Narcea sampling locations or the tributaries of the Tâmega sub-basin of the Douro basin (Table S3 in Supporting information). Low F_ST_ values were also found between the Alberche river (Tagus basin) and most of the remaining populations, and between the Porcia Cantabrain basin and other Cantabrian (Eo and Ouro) and Atlantic basins (Tambre and Ulla). However, Alberche samples had a relatively high proportion of missing data in comparison to other populations, therefore this result has to be taken with caution. The higher F_ST_ values were found between pairwise comparisons of either Esva and Esqueiro Cantabrian basins with several Douro basin populations, as well as between the Tâmega sub-basin (Douro) and Atlantic-Cantabrian populations (Tables 2 and S3 in Supporting information). Very little differentiation was found between streams belonging to the same sub-basin in the Douro watershed, particularly in the Tâmega sub-basin where no differentiation was detected between specimens from distinct streams (Table S3 in Supporting information).

**Table 2:** F_ST_ pairwise comparisons at sub-basin level based on the Weir and Cockerham’s (below diagonal) estimator.

		Cantabrian							Atlantic					Douro			Tagus
		Narcea	Esqueiro	Esva	Porcia	Navia	Eo	Ouro	Mandeo	Tambre	Ulla	Bibey	Neiva	Tamega	Tuela	Paiva	Alberche
Cantabrian	Narcea		**0.08**	**0.05**	**0.04**	**0.08**	**0.06**	**0.03**	**0.07**	**0.04**	**0.05**	**0.05**	**0.09**	**0.11**	**0.1**	**0.09**	**0.006**
	Esqueiro			0.09	0.07	− 0.06	0.08	0.06	0.05	0.04	**0.07**	0.08	0.1	**0.13**	**0.11**	**0.12**	0.02
	Esva				− 0.03	0.09	0.05	0.03	0.07	0.04	**0.05**	0.07	0.11	0.13	**0.1**	**0.14**	0.03
	Porcia					0.05	− 0.04	− 0.02	0.04	0.02	0.04	0.07	0.07	0.12	0.08	0.11	− 0.004
	Navia						0.07	0.04	0.04	0.03	0.06	− 0.002	0.09	**0.03**	**0.1**	**0.11**	− 0.002
	Eo							− 0.005	0.05	0.006	**0.04**	0.05	0.07	**0.1**	**0.07**	**0.1**	− 0.04
	Ouro								0.03	0.02	0.04	0.02	0.05	**0.08**	**0.06**	**0.08**	− 0.05
Atlantic	Mandeo									0.02	**0.04**	0.06	0.08	**0.11**	**0.09**	**0.1**	0.0008
	Tambre										0.02	0.03	0.06	**0.07**	**0.04**	**0.08**	− 0.04
	Ulla											0.01	**0.07**	**0.07**	**0.07**	**0.09**	− 0.06
	Bibey												0	**0.09**	**0.06**	**0.08**	0.04
	Neiva													**0.07**	**0.04**	**0.08**	0.001
Douro	Tamega														**0.04**	**0.1**	**0.06**
	Tuela															**0.09**	**0.003**
	Paiva																**0.02**
Tagus	Alberche																

In bold significant values at 95% confidence level for the Weir and Cokerham’s estimator after Bonferroni (p-value: 0.003) corrections.

Although the AMOVA analyses showed the highest percentage of explained genetic variation (94.01%) among individuals within locations, indicating overall limited genetic differentiation, there was an important percentage of variation attributed to differences between hydrological sub-basins (8.46%, p < 0.05; Table S4 in Supporting Information). This is consistent with the moderate estimated pairwise F_ST_ (> 0.10 for many comparisons), statistically significant at both location and basin/sub-basin, especially those involving the Douro lineage with the remaining populations belonging to the Atlantic and Cantabrian lineages, as well as the Tagus Basin (Alberche). These results indicate a high differentiation of Douro populations (Table 2). Additionally, none of the F_ST_ pairwise comparisons involving the Porcia Cantabrian basin were significant (Table 2). The Narcea Cantabrian basin was statistically significantly different from all the other basins/sub-basins, with FST values larger than 0.05 for most comparisons (Table 2).

**Figure 2:**
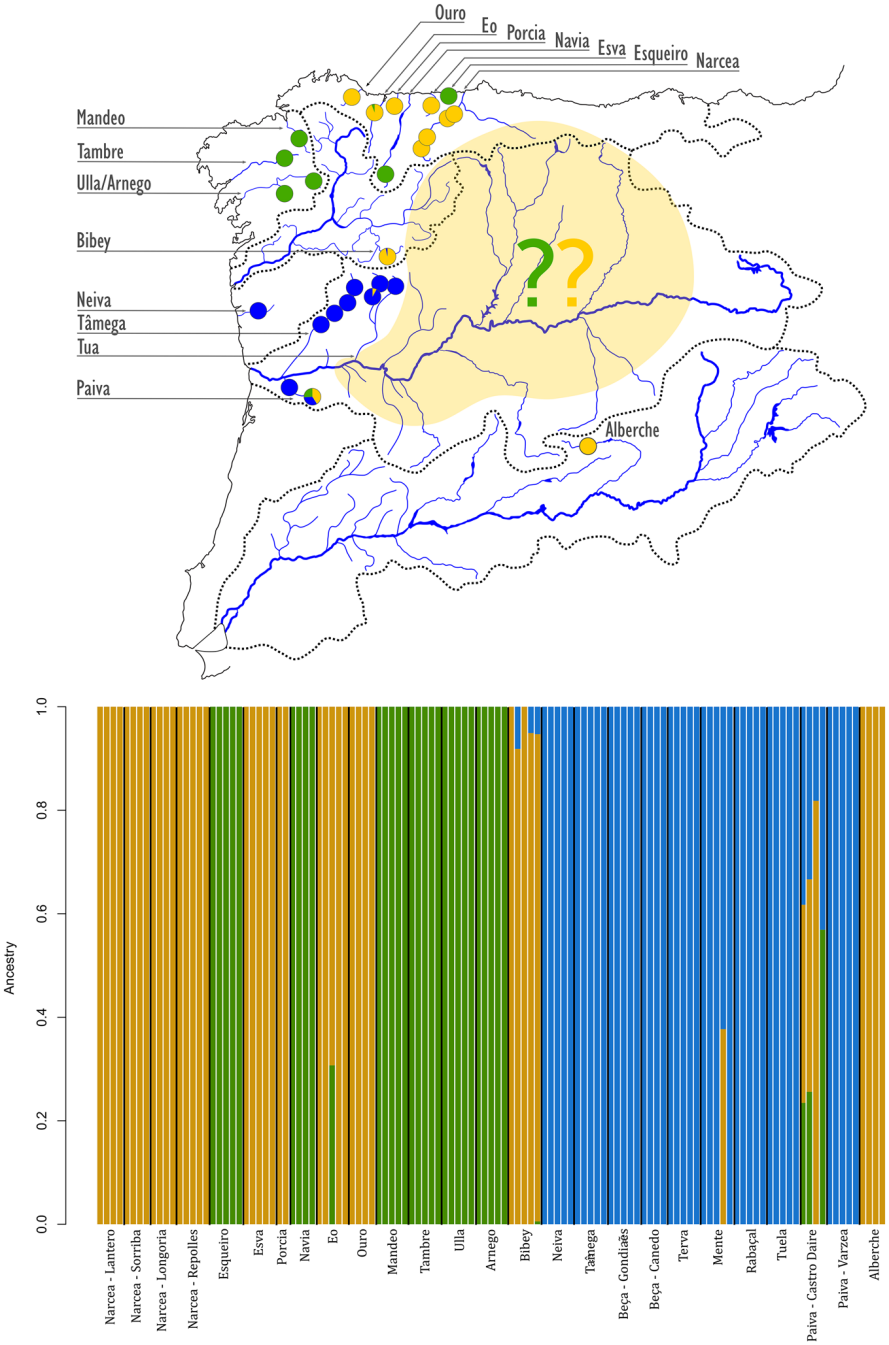
Ancestral populations contribution in each location considering K = 3 based on Admixture software.

The sNMF and Admixture results were mostly consistent regarding the assignment of individuals to each cluster (Fig. S1). However, the methodologies differed regarding the most plausible number of clusters according to cross-entropy (sNMF), where K = 3 had the lowest cross-entropy followed by K = 2, and cross-validation error (Admixture), where K = 1 had the lowest cross-validation error, followed by K = 2 and K = 3 (Fig. S2 in Supporting Information). For K = 2, the Atlantic and Cantabrian basins were clustered together with the Alberche population (Tagus basin), whereas the other cluster included the Douro basin populations along with the Portuguese Neiva small coastal basin (referred to hereafter as “Douro lineage”) (Fig. S1 in Supporting Information). When using K = 3, the Atlantic—Cantabrian—Alberche group was subdivided into two differentiated clusters: one of them was mainly formed by the Atlantic populations (referred to hereafter as “Atlantic lineage”) but including the Esqueiro and Navia basins located in the eastern Cantabrian slope; the second one was constituted by the Cantabrian populations (referred to hereafter as “Cantabrian lineage”), which also grouped the Bibey (Minho Basin) and the Alberche (Tagus Basin) populations (Fig. 2). The Paiva River, a tributary of the left bank of the Douro Basin, showed an even mixture of the three genetic lineages in the sampling location closer to its source, while all the individuals from the downstream location in this river were assigned to the Douro lineage. Likewise, individuals with contributions from different clusters were found in the Eo Basin (Atlantic and Cantabrian lineages); in the Bibey sub-basin (Minho basin) and the Tuela river (Douro basin) (Cantabrian and Douro lineages) (Fig. 2).

### Association between genetic and geographic distances

**Figure 3:**
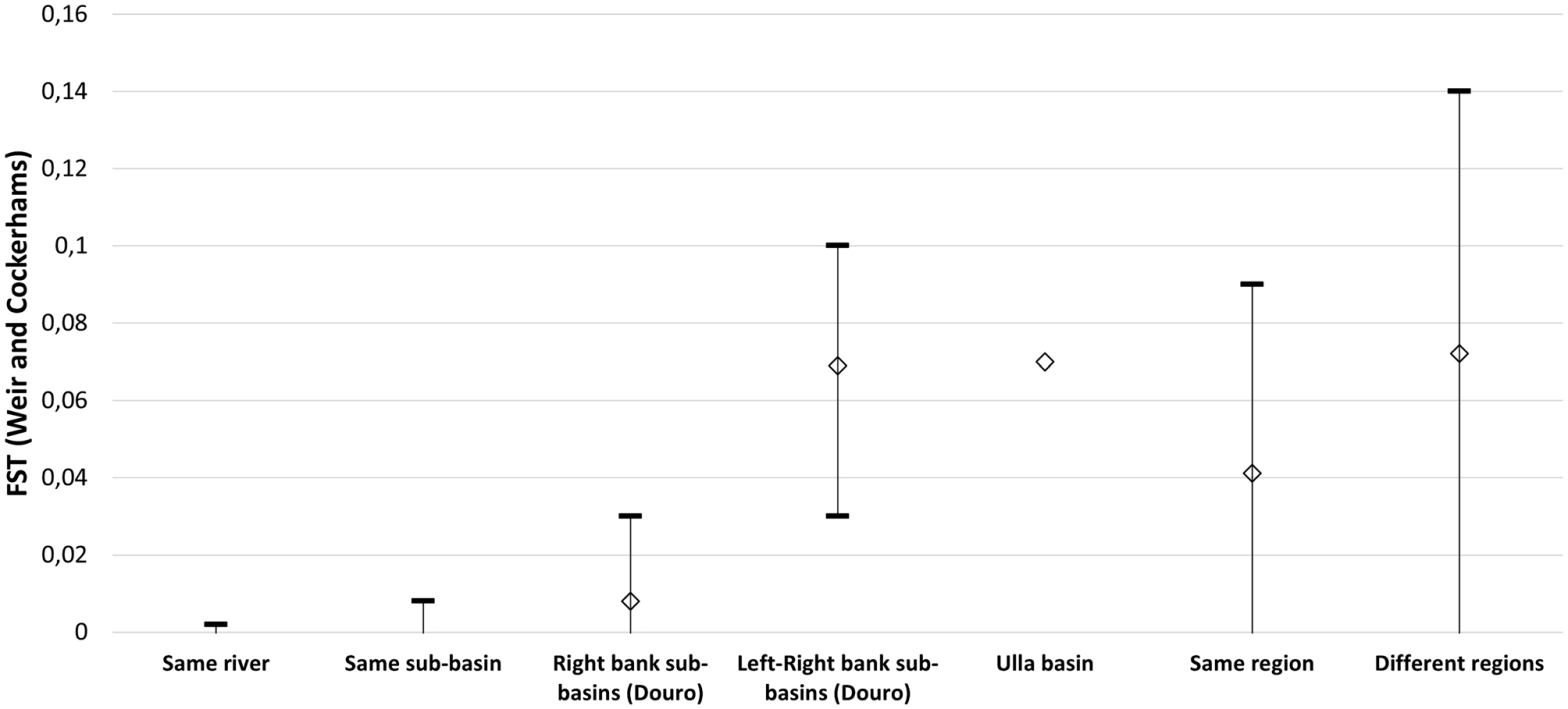
Range of F_ST_ values (Weir and Cockerham’s estimator) between pairs of locations for each hydrologic unit category, considering “region” as Atlantic, Cantabrian and Douro basins.

**Figure 4:**
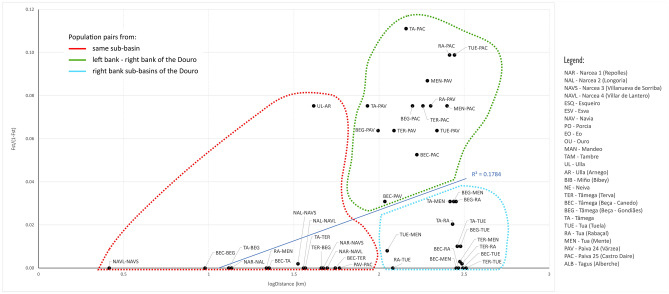
Relation between F_ST_ values (Weir and Cockerham’s estimator) and linear geographic distance between pairs of locations within the same basin (Douro, Ulla and Narcea).

In general, the range of F_ST_ values increased when considering pairwise comparisons in the following sequence: within the same river, within the same sub-basin, between sub-basins of the right bank of the Douro river, between sub-basins on either bank of the Douro river, and between major regions (Atlantic, Cantabrian and Douro) (Fig. 3). Within river basins we found higher F_ST_ values between locations showing higher linear distances between them (Fig. 4); however, for the same linear distance, Fst values were always higher between locations on either margin of the Douro river (green in Fig. 4) than on the same margin (blue on Fig. 4). On the other hand, Fst values were exceptionally high between the Ulla river and its tributary Arnego considering the linear distance between them.

## Discussion

Conservation units from a genetic point of view should be regarded as having a common ancestor and evolutionary history, as well as enough genetic diversity, so that adaptation to local conditions and adaptability to future changes are adequately balanced^8^. Highly threatened populations that show considerable genetic diversity depletions are often subjected to recovery plans which include conservation measures like the injection of genetic novelty, through the introduction of specimens from other populations, and the increase of population size to minimize stochastic threats^42^. However, genetic data has seldom been considered in *M. margaritifera* conservation actions, even though it is a highly threatened species with multiple captive breeding and restocking programs throughout Europe^18,43^.

Our SNP data corroborate previous studies based on microsatellites that found low genetic diversity in Iberian hydrological basins^20,21^. However, in these studies the Ulla (Arnego) and Eo populations (respectively, from the Atlantic and Cantabrian slopes of Iberia) exhibited low values of genetic diversity, which was not the case in the present study. These inconsistencies may be associated with the lower number of loci analyzed in microsatellites studies when compared to SNP datasets, hence SNPs reflect genome-wide genetic diversity of populations more accurately than microsatellites^6^. The overall low genetic diversity values found for *M. margaritifera* are commonly found in other endangered freshwater organisms, such as Australian freshwater crocodiles and Mexican golden trout^44,45^ and have also been estimated for North American *M. margaritifera*^24^ and *M. hembeli*^26^, suggesting a strong relation between the loss of genetic diversity and the poor conservation status of these species. The low genetic diversity values found in the Iberian populations have been explained by strong genetic drift effects due to population bottlenecks^20,21^. Populations with low effective population sizes face stronger effects of genetic drift, leading to a random loss or fixation of alleles, and consequently resulting in the loss of genetic diversity^42^. Nevertheless, some of the largest Iberian populations (census data) such as that of the Narcea Cantabrian river showed the lowest diversity, whereas some of the populations with the smallest census data such as that from the Neiva small Atlantic river basin showed higher diversity values. Although genetic diversity was low, no evidence of significant inbreeding was found for any *M. margaritifera* populations (FIS values close to zero). This was an unexpected result considering the high autofecundation described for some of the Iberian populations^20^, which is a common strategy in freshwater mussels when environmental conditions are not optimal or when population sizes are small^46^ and may be a methodological artifact.

Previous studies based on microsatellites showed a strong structure among European *M. margaritifera* populations, which has been associated to the effect of genetic drift and founder effects in isolated populations, ecological differentiation due to different selective pressures, or to the human impact that have led to habitat fragmentation and decreasing of gene flow^20–23^. Contrary to these findings, mitochondrial markers (COI and 16S rRNA) or allozyme data have not supported such levels of population differentiation^47^, probably because of slower evolutionary rates of these markers relative to microsatellites. Therefore, our results confirm and complement previous studies based on microsatellites and show a strong differentiation between populations from different basins and sub-basins, but variable levels of differentiation within sub-basins. Even so, several pairs of populations from the Atlantic and Cantabrian coasts show very little differentiation, suggesting they may have been separated recently (e.g. by tectonic events leading to stream capture between basins, as it is known to have occurred in the studied area^48^). Our results concerning the Douro River system and the small independent Atlantic basins to the north, such as the Neiva river, indicate that gene flow between these populations was maintained until more recently than between Atlantic and Cantabrian populations. The low or neglectable differentiation within the same river or sub-basin suggests that gene flow was effective across locations. On the other hand, the higher differentiation detected between populations from either banks of the river Douro suggests that the main course of this river constituted an effective barrier to gene flow. This may be related to migratory paths and behavior of host fish in the past.

In peripheral distribution ranges, as is the case of the Iberian *M. margaritifera* populations, higher genetic differentiation and low levels of genetic diversity are expected due to the presence of suboptimal or atypical habitat conditions in comparison to core populations^49^. The geographical position of the Iberian Peninsula, located under the influence of the Mediterranean climate, constitutes the southern limit of several European freshwater organisms, which exhibit such genetic patterns (e.g. *Salmo trutta*^50^; *Gasterosteus aculeatus*^51^). It is hypothesized that these species could not extend their distribution to Northern Africa due to either climatic (more arid Mediterranean climate conditions^52^) or biogeographical (Gibraltar Strait as a strong biogeographic barrier since the last 5.3 Ma^53^) constraints; nevertheless, in the Iberian Peninsula, these species are often restricted to sparse areas that are ecologically adequate, probably leading to local adaptation processes.

**Figure 5:**
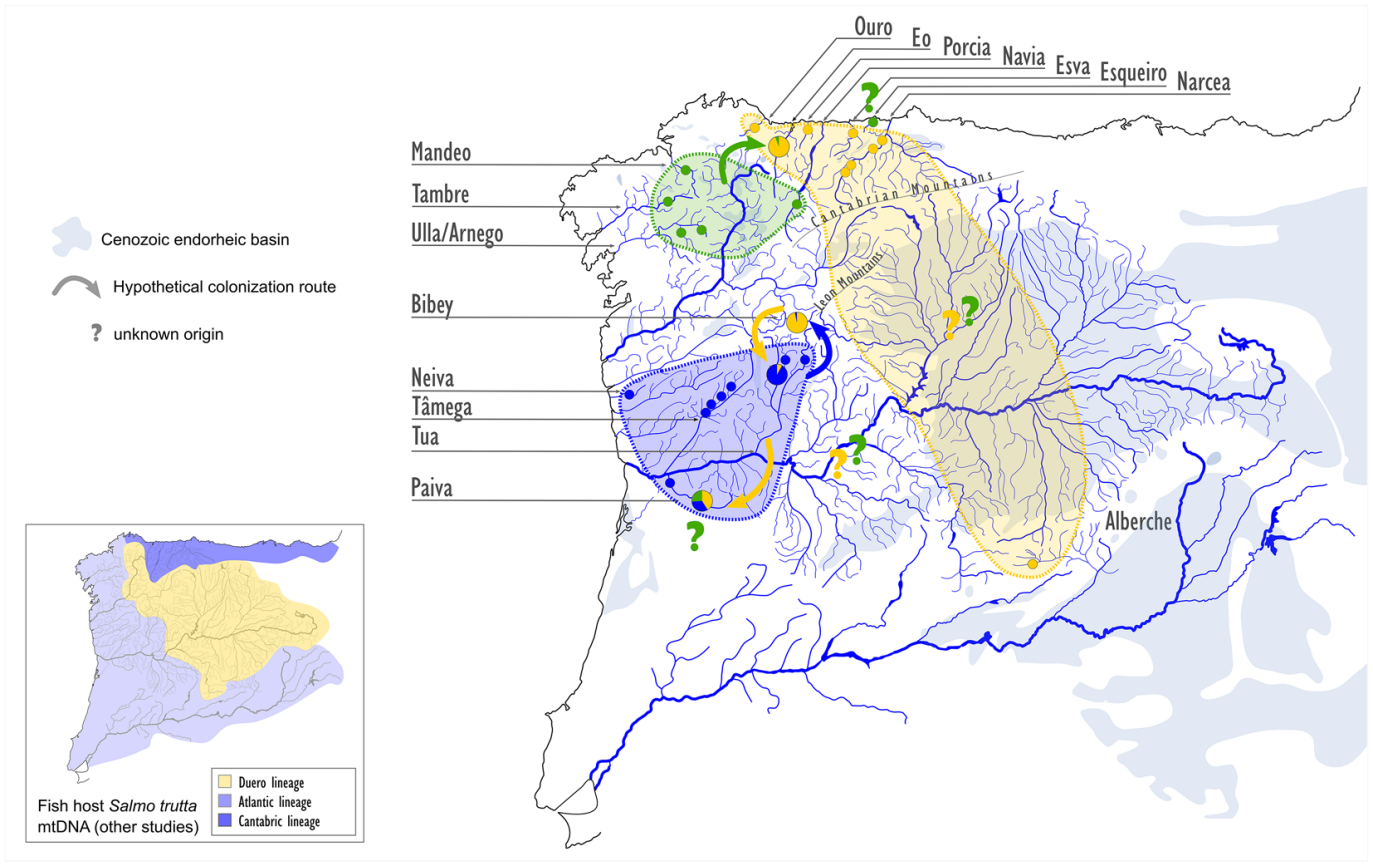
Hypothetical colonization routes of *Margaritifera margaritifera* in the Iberian Peninsula in relation to genetic population structure of its host *Salmo trutta* (lower left corner).

The pattern of high differentiation that we found for the Iberian *M. margaritifera* populations in our study reflects, among other factors, the dispersal abilities of mussel species and their fish hosts^54,55^. A strong relation between the degree of genetic distinctiveness and the fish host species has been confirmed for *M. margaritifera*: the genetic structure is more intensely marked when the fish host is *Salmo trutta* (trout) rather than *Salmo salar* (Atlantic salmon), due to the higher dispersal ability of the Atlantic salmon^56,57^. In the Iberian Peninsula, the strong decline of the Atlantic salmon^15^ often left the trout as the only available host for most *M. margaritifera* populations. In addition, the genetic structure of the Iberian brown trout is in part congruent with our results for the genetic structure of Iberian *M. margaritifera,* implying some degree of co-evolution between the two species: three main trout lineages (Atlantic, Douro and Mediterranean) have been recognized and associated to the role of Iberian glacial refuges in the Pleistocene^58,59^. The Atlantic lineage actually includes the populations closer to the lower course of the Douro river, apart from populations from Galician and Cantabrian coastal basins, effectively splitting the Douro basin trout populations in two separate lineages (Fig. 5). The presence of those two trout genetic lineages in the Douro Basin resembles the genetic differentiation between downstream and upstream Douro sub-basins for *M. margaritifera* populations described by^21^. Furthermore, the Atlantic trout lineage is subdivided into two well-supported sub-lineages, a North-Atlantic sub-lineage that includes Galician and Cantabrian basins and a South-Atlantic lineage that includes the western Douro Basin^58,59^, which is consistent with the separation of the western Douro from the Atlantic and Cantabrian lineages of *M. margaritifera*. In the Tagus Basin, both the Atlantic and eastern Douro lineages of trout are present, and it has been hypothesized that the genotypes from the Douro basin reached the Tagus through river captures^58,59^. Indeed, studies based on mitochondrial DNA have revealed the presence of common haplotypes in Cantabrian, Douro and Tagus basins, indicating past connections between these areas^58^, which may explain the close relationship of the *M. margaritifera* population from the Alberche river with the Cantabrian lineage. Therefore, the presence of the Cantabrian lineage in the Alberche, Bibey and Paiva populations may suggest that probably, in the past, *M. margaritifera* was widely distributed to the north of the Iberian central mountainous system (Fig. 5) and that populations from the eastern Douro basin not analyzed in this study also belong to this lineage. Indeed, the recent discovery of a relict population close to the source of the river Douro seems to support this hypothesis^60^. This congruent drainage-specific genetic structure pattern between *M. margaritifera* and its brown trout host has also been described in other European populations^61^.

Peripheral populations have a high importance from a conservation point of view, as they have been considered as potential reservoirs of adaptive genetic variation, even if they usually retain low genetic diversity^62^. It is a matter of fact that genetic diversity is the basis of evolutionary change and, consequently, it is decisive for organisms to adapt to changing environmental conditions under the current global warming scenario, especially when the potential impact is significant, as is the case of *M. margaritifera*^16^. Even though our study supports a high differentiation among populations, it does suggest the occurrence of recent gene flow events, either through the fluvial connectivity within rivers and basins, or through past paleogeomorphological/tectonic events such as river captures. This underscores the importance of preserving the whole diversity of Iberian populations as they reflect evolutionary pathways that are intimately related to the transition to exorheism of Iberian paleo-basins in the last 5 My, and in straight relation with its preferred Iberian host, the brown trout. Habitat fragmentation due to dam construction is a widespread threat for *M. margaritifera* and a further obstacle for gene flow, but its impacts were not addressed in this study. Habitat fragmentation imposed by dams is thought to have been responsible for the extinction of the Atlantic salmon in some Atlantic and Cantabrian rivers^63,64^. The limitation of host movements has a direct impact on the mussel’s capacity to maintain gene flow along the river, and at the present time, Iberian *M. margaritifera* populations are spatially distributed in a fragmented way and with reduced abundances.

Genetic structure and genetic diversity are gaining more importance in the development of more effective conservation plans, as both are considered key components of biological diversity^65^, and therefore, any conservation action proposed for the preservation of *M. margaritifera* should take these parameters into account. Indeed, the combination of ecological and genetic studies may greatly benefit the design of accurate conservation plans (e.g.^66^), which are urgently needed for *M. margaritifera* populations to prevent imminent extinction. Considering the results of genetic structure overall, at least three different conservation units could be identified in the Iberian Peninsula for *M. margaritifera,* based on the three large genetic lineages: Atlantic, Cantabrian and western Douro. However, due to the singularity of each independent population within the three genetic lineages and the low genetic diversity found in most of them, any planned conservation actions must take in consideration the specific genetic patterns of each population. Our study showed that, in some cases, several tributaries share the same genetic identity and can be considered a single population, but in other cases different genetic backgrounds are identified within the same river. This emphasizes the importance of not generalizing results and accurately characterizing each population before planning conservation actions. We can therefore identify conservation units for *M. margaritifera* in the Iberian Peninsula at different scales: a large single metapopulation that includes all the species distribution and that had the potential to maintain gene flow to different degrees through river continuity or geologic events; regional units (Atlantic, Cantabrian and Douro), sub-basin (e.g. Tâmega sub-basin), river (e.g. Ulla, Arnego) or location (e.g. Paiva). The identification of these discrete evolutionary units is crucial for species conservation, as it helps to define management units to best protect genetic diversity without eroding genetic structure^67^.

*M. margaritifera* has been the object of much conservation interest in Europe for the last two decades^9,68^. Several *in-situ* and *ex-situ* conservation actions have been proposed and implemented throughout the continent, including the Iberian Peninsula^13^. Captive breeding programs may be implemented to restock or reintroduce the species in a certain river system^68–70^ and have been extensively implemented through LIFE projects. Restocking and reintroduction may also be achieved via translocation within or between river systems^71^. The translocation of specimens within a river system is a common action to bring together the few specimens left in certain patches and, in this way, increase the probability of fertilization and glochidia production, or to avoid the negative impact of local negative pressures, such as bridge construction. A river or stream is often used as a basic conservation unit for freshwater mussels. However, our results suggest that the adequate unit to minimize genetic mixture may be a whole sub-basin/basin (as is the case of the Tâmega sub-basin). In other cases, some genetic structure is present within the same river, such as in the Paiva and Narcea rivers, which may lead to a narrower definition of conservation unit. In these cases, translocations may be difficult to plan to preserve the original gene pools, as it requires in depth study of the species genetic structure along the river.

It can also be argued that under the current situation where most populations are isolated, reduced or both, translocating mussels from different areas of the same river systems may contribute to increase genetic diversity, much like the free movement of hosts would do in undisturbed conditions, and thus favoring the conservation prospects of the species. In this sense, translocations between rivers, sub-basins or even basins, in theory, would potentially benefit the species ability to cope with changes. However, balancing the maintenance of evolutionary genetic identity and gene flow across river systems is a delicate process that must be carefully planned case-by-case in a framework that considers all possible outputs. Furthermore, possible habitat adaptations of specific genetic profiles may also have evolved. Discarding this possibility may jeopardize altogether the success of a translocation or reintroduction program. The inclusion of more individuals per population or different analytical methodologies such as whole genome resequencing could help reducing the limitations from our data set, such as the presence of missing data or the number of individuals (two in Porcia) and allow taking sound conservation decisions.

The understanding of the genetic structure and diversity of *M. margaritifera* populations in the Iberian Peninsula shed some light on relevant issues in planning and development of effective management decisions, including restocking and translocations. Indeed, an integrative conservation approach combining genetic and ecological knowledge about the species is urgently needed for the long-term preservation of the freshwater pearl mussel.

## Supplementary Information

Supplementary Information.

## Data Availability

All the data files are available from the National Center for Biotechnology Information (NCBI) Sequence-Read Archive (SRA) database with the accession numbers: SRR21493896-SRR21494025 (Bioproject PRJNA876830). Final vcf file and custom scripts used for some analyses have been released in the figshare repository under the link https://figshare.con/s/ba04242816afcde74cd1.
