# Supplementary material for: Applying genomic approaches to delineate conservation strategies using the freshwater mussel Margaritifera margaritifera in the Iberian Peninsula as a model
Source: Sci Rep. 2022 Oct 7;12:16894. doi: 10.1038/s41598-022-20947-5 (PMC9546909; doi:10.1038/s41598-022-20947-5)
Supplement: Supplementary file 1 — Supplementary Information. [file 41598_2022_20947_MOESM1_ESM.docx]

**Table S1.** Evolutionary lineages; sampling localities; number of sampled and analysed individuals per population in parenthesis; sample names (vouchers), in bold samples used to build the catalog in stacks (M1m4n2, 1 individual/sub-basin); GenBank Accession numbers (Biosample Accesion); % missing data per sample before SNP-filtering; Mean depth coverage per sample.

| **Lineage** | **Basin** | **Sub-basin** | **River/Sampling location (number of sampled/analyzed individuals after SNP-filtering)** | **Locality** | **Sample** | **GenBank Accession Number (BioSample)** | **% missing data** | **Mean depth coverage** |
| --- | --- | --- | --- | --- | --- | --- | --- | --- |
| **Cantabrian** | Narcea | | Narcea 1 (5/5) | Repolles | **MNR_2** | SAMN30673506 | 19.23 | 16.91 |
|  |  |  |  |  | MNR_3 | SAMN30673507 | 42.89 | 8.89 |
|  |  |  |  |  | MNR_4 | SAMN30673508 | 27.27 | 8.08 |
|  |  |  |  |  | MNR_5 | SAMN30673509 | 32.53 | 9.06 |
|  |  |  |  |  | MNR_6 | SAMN30673510 | 38.10 | 8.41 |
|  |  |  | Narcea 2 (5/4) | Longoria | MNL_2 | SAMN30673511 | 41.13 | 8.01 |
|  |  |  |  |  | MNL_4 | SAMN30673512 | 53.52 | 6.48 |
|  |  |  |  |  | MNL_5 | SAMN30673513 | 75.36 | 7.98 |
|  |  |  |  |  | MNL_6 | SAMN30673514 | 47.40 | 5.42 |
|  |  |  |  |  | MNL_7 | SAMN30673515 | 26.50 | 11.58 |
|  |  |  | Narcea 3 (5/4) | Villanueva de Sorriba | MNS_1 | SAMN30673516 | 85.38 | 4.50 |
|  |  |  |  |  | MNS_2 | SAMN30673517 | 45.38 | 6.15 |
|  |  |  |  |  | MNS_3 | SAMN30673518 | 35.80 | 12.80 |
|  |  |  |  |  | MNS_4 | SAMN30673519 | 29.24 | 10.28 |
|  |  |  |  |  | MNS_6 | SAMN30673520 | 25.25 | 9.88 |
|  |  |  | Narcea 4 (5/4) | Villar de Lantero | MNV_1 | SAMN30673521 | 60.44 | 7.97 |
|  |  |  |  |  | MNV_2 | SAMN30673522 | 53.32 | 10.67 |
|  |  |  |  |  | MNV_4 | SAMN30673523 | 27.30 | 9.22 |
|  |  |  |  |  | MNV_5 | SAMN30673524 | 51.96 | 8.14 |
|  |  |  |  |  | MNV_6 | SAMN30673525 | 41.19 | 7.53 |
|  | Esqueiro | | Esqueiro (5/5) | San Pedro de la Ribera | MEPR_1 | SAMN30673526 | 37.62 | 7.82 |
|  |  |  |  |  | MEPR_2 | SAMN30673527 | 35.91 | 10.99 |
|  |  |  |  |  | **MEPR_3** | SAMN30673528 | 21.49 | 12.07 |
|  |  |  |  |  | MEPR_4 | SAMN30673529 | 25.58 | 10.45 |
|  |  |  |  |  | MEPR_5 | SAMN30673530 | 37.62 | 8.12 |
|  | Esva | | Esva (5/5) | Meres | **MEM_1** | SAMN30673531 | 47.78 | 11.60 |
|  |  |  |  |  | MEM_2 | SAMN30673532 | 28.15 | 10.15 |
|  |  |  |  |  | MEM_4 | SAMN30673533 | 30.81 | 10.38 |
|  |  |  |  |  | MEM_5 | SAMN30673534 | 25.39 | 7.38 |
|  |  |  |  |  | MEM_6 | SAMN30673535 | 27.40 | 8.73 |
|  | Porcia | | Porcia (5/2) | Ermita de los Remedios | **MPR_1** | SAMN30673536 | 27.80 | 6.21 |
|  |  |  |  |  | MPR_4 | SAMN30673537 | 66.69 | 4.16 |
|  |  |  |  |  | MPR_5 | SAMN30673538 | 32.61 | 11.90 |
|  |  |  |  |  | MPR_6 | SAMN30673539 | 88.21 | 9.57 |
|  |  |  |  |  | MPR_7 | SAMN30673540 | 67.68 | 6.21 |
|  | Navia | | Navia (5/4) | Evernalla | **MNE_1** | SAMN30673541 | 25.30 | 11.11 |
|  |  |  |  |  | MNE_2 | SAMN30673542 | 42.87 | 6.77 |
|  |  |  |  |  | MNE_3 | SAMN30673543 | 22.40 | 11.32 |
|  |  |  |  |  | MNE_4 | SAMN30673544 | 24.42 | 5.99 |
|  |  |  |  |  | MNE_6 | SAMN30673545 | 65.52 | 12.50 |
|  | Eo | | Eo (5/5) | Trasdacorda | **MET_1** | SAMN30673546 | 27.54 | 10.17 |
|  |  |  |  |  | MET_2 | SAMN30673547 | 22.35 | 9.48 |
|  |  |  |  |  | MET_3 | SAMN30673548 | 38.20 | 7.95 |
|  |  |  |  |  | MET_4 | SAMN30673549 | 25.59 | 13.10 |
|  |  |  |  |  | MET_6 | SAMN30673550 | 31.06 | 6.48 |
|  | Ouro | | Ouro (5/4) | Castro de Ouro | **MOU_1** | SAMN30673551 | 26.31 | 9.28 |
|  |  |  |  |  | MOU_2 | SAMN30673552 | 67.71 | 12.08 |
|  |  |  |  |  | MOU_3 | SAMN30673553 | 37.92 | 7.95 |
|  |  |  |  |  | MOU_4 | SAMN30673554 | 26.59 | 13.10 |
|  |  |  |  |  | MOU_5 | SAMN30673555 | 31.06 | 6.48 |
|  | Tagus | Alberche | Alberche (5/4) | Burgohondo | **MAL_1** | SAMN30673556 | 50.95 | 3.89 |
|  |  |  |  |  | MAL_2 | SAMN30673557 | 81.77 | 7.87 |
|  |  |  |  |  | MAL_3 | SAMN30673558 | 56.59 | 7.70 |
|  |  |  |  |  | MAL_5 | SAMN30673559 | 54.17 | 7.38 |
|  |  |  |  |  | MAL_6 | SAMN30673560 | 52.62 | 6.61 |
| **Atlantic** | Mandeo | | Mandeo (5/5) | O Sisto | **MMdeo_1** | SAMN30673561 | 30.68 | 14.32 |
|  |  |  |  |  | MMdeo_2 | SAMN30673562 | 33.64 | 10.38 |
|  |  |  |  |  | MMdeo_3 | SAMN30673563 | 34.63 | 12.10 |
|  |  |  |  |  | MMdeo_4 | SAMN30673564 | 26.75 | 9.67 |
|  |  |  |  |  | MMdeo_5 | SAMN30673565 | 24.48 | 16.30 |
|  | Tambre | | Tambre (5/5) | Ponte Carreira | **MTamb_1** | SAMN30673566 | 37.04 | 14.90 |
|  |  |  |  |  | MTamb_2 | SAMN30673567 | 24.96 | 11.22 |
|  |  |  |  |  | MTamb_3 | SAMN30673568 | 20.62 | 12.69 |
|  |  |  |  |  | MTamb_4 | SAMN30673569 | 36.50 | 8.19 |
|  |  |  |  |  | MTamb_5 | SAMN30673570 | 29.32 | 9.89 |
|  | Ulla | Ulla | Ulla (5/5) | Antas de Ulla | **A_720** | SAMN30673571 | 18.81 | 13.04 |
|  |  |  |  |  | A_721 | SAMN30673572 | 15.20 | 10.25 |
|  |  |  |  |  | A_727 | SAMN30673573 | 26.60 | 20.42 |
|  |  |  |  |  | A_730 | SAMN30673574 | 21.19 | 9.56 |
|  |  |  |  |  | A_734 | SAMN30673575 | 18.08 | 12.52 |
|  |  | Arnego | Arnego (5/5) | Vila de Cruces | A_518 | SAMN30673576 | 21.32 | 9.94 |
|  |  |  |  |  | A_522 | SAMN30673577 | 22.52 | 10.18 |
|  |  |  |  |  | C_018 | SAMN30673578 | 25.68 | 10.41 |
|  |  |  |  |  | C_019 | SAMN30673579 | 31.91 | 9.96 |
|  |  |  |  |  | C_021 | SAMN30673580 | 32.92 | 9.48 |
|  | Miño | Bibey | Bibey (5/5) | San Sebastián | **MBib_1** | SAMN30673581 | 52.42 | 8.58 |
|  |  |  |  |  | MBib_2 | SAMN30673582 | 39.60 | 8.40 |
|  |  |  |  |  | MBib_3 | SAMN30673583 | 24.67 | 11.14 |
|  |  |  |  |  | MBib_4 | SAMN30673584 | 22.75 | 6.93 |
|  |  |  |  |  | MBib_5 | SAMN30673585 | 50.78 | 6.91 |
|  | Neiva | | Neiva (5/5) | Balugães | **MNe_2** | SAMN30673586 | 34.34 | 10.05 |
|  |  |  |  |  | MNe_2 | SAMN30673587 | 32.22 | 9.53 |
|  |  |  |  |  | MNe_2 | SAMN30673588 | 32.10 | 10.59 |
|  |  |  |  |  | MNe_2 | SAMN30673589 | 41.39 | 10.67 |
|  |  |  |  |  | MNe_2 | SAMN30673590 | 34.49 | 11.42 |
| **Douro** | Douro | Tâmega | Tâmega (5/5) | Cavês | **Mta_1** | SAMN30673591 | 36.38 | 10.49 |
|  |  |  |  |  | Mta_2 | SAMN30673592 | 43.77 | 10.78 |
|  |  |  |  |  | Mta_3 | SAMN30673593 | 32.21 | 10.59 |
|  |  |  |  |  | Mta_4 | SAMN30673594 | 41.39 | 10.67 |
|  |  |  |  |  | Mta_5 | SAMN30673595 | 34.49 | 11.42 |
|  |  |  | Beça G (5/5) | Gondiães | MBG_1 | SAMN30673596 | 38.12 | 9.87 |
|  |  |  |  |  | MBG_2 | SAMN30673597 | 35.81 | 9.35 |
|  |  |  |  |  | MBG_3 | SAMN30673598 | 32.88 | 10.42 |
|  |  |  |  |  | MBG_4 | SAMN30673599 | 39.81 | 10.27 |
|  |  |  |  |  | MBG_5 | SAMN30673600 | 33.45 | 9.12 |
|  |  |  | Beça C (5/5) | Canedo | MBC_1 | SAMN30673601 | 42.66 | 9.87 |
|  |  |  |  |  | MBC_2 | SAMN30673602 | 42.22 | 8.10 |
|  |  |  |  |  | MBC_3 | SAMN30673603 | 42.12 | 7.85 |
|  |  |  |  |  | MBC_4 | SAMN30673604 | 69.22 | 5.98 |
|  |  |  |  |  | MBC_5 | SAMN30673605 | 48.39 | 10.07 |
|  |  |  | Terva (5/5) | Boticas | MTr_1 | SAMN30673606 | 41.05 | 10.48 |
|  |  |  |  |  | MTr_2 | SAMN30673607 | 43.72 | 9.84 |
|  |  |  |  |  | MTr_3 | SAMN30673608 | 37.51 | 10.86 |
|  |  |  |  |  | MTr_4 | SAMN30673609 | 53.78 | 8.77 |
|  |  |  |  |  | MTr_5 | SAMN30673610 | 46.49 | 6.96 |
|  |  | Tua | Mente (5/5) | São Jomil | MMJ_2 | SAMN30673611 | 36.87 | 9.72 |
|  |  |  |  |  | MMJ_3 | SAMN30673612 | 41.20 | 10.96 |
|  |  |  |  |  | MMJ_4 | SAMN30673613 | 38.93 | 10.41 |
|  |  |  |  |  | MMJ_5 | SAMN30673614 | 35.73 | 9.96 |
|  |  |  |  |  | MMJ_7 | SAMN30673615 | 26.50 | 11.43 |
|  |  |  | Rabaçal | Revelhe | MRR_1 | SAMN30673616 | 46.05 | 10.55 |
|  |  |  |  |  | MRR_2 | SAMN30673617 | 42.62 | 10.08 |
|  |  |  |  |  | MRR_3 | SAMN30673618 | 34.22 | 9.02 |
|  |  |  |  |  | MRR_4 | SAMN30673619 | 39.25 | 10.60 |
|  |  |  |  |  | MRR_5 | SAMN30673620 | 39.53 | 9.60 |
|  |  |  | Tuela | Fresulfe | MTF_1 | SAMN30673621 | 29.39 | 10.72 |
|  |  |  |  |  | **MTF_2** | SAMN30673622 | 32.70 | 9.32 |
|  |  |  |  |  | MTF_3 | SAMN30673623 | 36.41 | 11.27 |
|  |  |  |  |  | MTF_4 | SAMN30673624 | 34.70 | 9.76 |
|  |  |  |  |  | MTF_5 | SAMN30673625 | 38.71 | 10.35 |
|  |  | Paiva | Paiva V (5/5) | Várzea | **MPV_1** | SAMN30673626 | 29.39 | 10.72 |
|  |  |  |  |  | MPV_3 | SAMN30673627 | 32.70 | 9.32 |
|  |  |  |  |  | MPV_4 | SAMN30673628 | 36.41 | 11.27 |
|  |  |  |  |  | MPV_5 | SAMN30673629 | 39.19 | 10.78 |
|  |  |  |  |  | MPV_6 | SAMN30673630 | 33.95 | 8038 |
|  |  |  | Paiva C (5/4) | Castro Daire | MPCD_1 | SAMN30673631 | 25.66 | 6.93 |
|  |  |  |  |  | MPCD_2 | SAMN30673632 | 70.60 | 10.38 |
|  |  |  |  |  | MPCD_3 | SAMN30673633 | 49.48 | 11.05 |
|  |  |  |  |  | MPCD_5 | SAMN30673634 | 23.40 | 8.24 |
|  |  |  |  |  | MPCD_6 | SAMN30673635 | 25.58 | 10.10 |

**Table S2.** Basic genetic diversity statistics estimated by the *populations* program of STACKS. *Sites* is the number of sites that have data for a particular population; *Variant sites* is the number of those sites that are polymorphic in at least one population; *Private sites are alleles that are found only in a single populations*; *Polymorphic sites* is the number of sites that are polymorphic within a particular population; *% polymorphic sites* is the percentage of sites that are polymorphic considering the total number of sites that have data for a particular population; and FIS is the inbreeding coefficient.

| Population | Sites | Variant sites | Private sites | Polymorphic sites | % polymorphic sites | FIS | StdErr  FIS |
| --- | --- | --- | --- | --- | --- | --- | --- |
| Narcea 4 | 30831 | 30807 | 77 | 3435 | 11.14 | 0.0020 | 0.0053 |
| Narcea 3 | 31374 | 31348 | 81 | 3630 | 11.57 | -0.0002 | 0.0051 |
| Narcea 2 | 30958 | 30932 | 76 | 3800 | 12.28 | 0.0013 | 0.0053 |
| Narcea 1 | 31603 | 31577 | 104 | 4751 | 15.03 | 0.0019 | 0.0057 |
| Esqueiro | 31609 | 31583 | 105 | 501 | 15.87 | -0.0067 | 0.0055 |
| Esva | 31591 | 31565 | 122 | 4729 | 14.97 | -0.0054 | 0.0057 |
| Porcia | 29598 | 29579 | 35 | 2337 | 7.89 | 0.0038 | 0.0029 |
| Navia | 31499 | 31473 | 91 | 4451 | 14.13 | -0.0076 | 0.0049 |
| Eo | 31639 | 31614 | 133 | 5679 | 17.95 | -0.0037 | 0.0056 |
| Ouro | 31473 | 31447 | 114 | 4737 | 15.05 | 0 | 0.0049 |
| Mandeo | 31544 | 31520 | 114 | 4722 | 14.97 | -0.0025 | 0.006 |
| Tambre | 31583 | 31558 | 162 | 5577 | 17.66 | -0.0026 | 0.0058 |
| Ulla | 31562 | 31536 | 225 | 5465 | 17.31 | -0.0015 | 0.0052 |
| Arnego | 31560 | 31534 | 144 | 5490 | 17.39 | -0.0095 | 0.0057 |
| Bibey | 31354 | 31328 | 114 | 3924 | 12.51 | -0.0049 | 0.006 |
| Neiva | 31597 | 31572 | 97 | 5273 | 16.69 | -0.0119 | 0.0058 |
| Tâmega | 31357 | 31332 | 40 | 4526 | 14.43 | -0.0131 | 0.0064 |
| Beça (19) | 31481 | 31456 | 52 | 4534 | 14.40 | -0.0107 | 0.0062 |
| Beça (18) | 30631 | 30607 | 25 | 3508 | 11.45 | -0.0096 | 0.0055 |
| Terva | 31126 | 31102 | 52 | 4284 | 13.76 | -0.0132 | 0.0065 |
| Mente | 31546 | 31520 | 58 | 5163 | 16.37 | -0.0122 | 0.0061 |
| Rabaçal | 31151 | 31125 | 50 | 4435 | 14.24 | -0.0145 | 0.0064 |
| Tuela | 31439 | 31413 | 75 | 5040 | 16.03 | -0.0137 | 0.0062 |
| Paiva (25) | 31465 | 31439 | 79 | 4107 | 13.05 | -0.0052 | 0.0049 |
| Paiva (24) | 31525 | 31500 | 56 | 4290 | 13.61 | -0.0101 | 0.006 |
| Alberche | 29406 | 29380 | 167 | 3025 | 10.29 | -0.0018 | 0.0053 |

Table S3. F_ST_ pairwise comparisons based on the Weir and Cockherhams’ estimator for all localities (above diagonal) and their p-values (below diagonal). In bold, significant p-values at 0.05 level

|  | **Alberche** | **Arnego** | **Beça Canedo** | **Beça Gondiaes** | **Bibey** | **Eo** | **Esqueiro** | **Esva** | **Mandeo** | **Mente** | **Narcea 1** | **Narcea 2** | **Narcea 3** | **Narcea 4** | **Navia** | **Neiva** | **Ouro** | **Paiva Castro Daire** | **Paiva Várzea** | **Porcia** | **Rabaçal** | **Tâmega** | **Tambre** | **Terva** | **Tuela** | **Ulla** |
| --- | --- | --- | --- | --- | --- | --- | --- | --- | --- | --- | --- | --- | --- | --- | --- | --- | --- | --- | --- | --- | --- | --- | --- | --- | --- | --- |
| **Alberche** | - | -0.04 | 0.06 | 0.04 | 0.04 | -0.04 | 0.02 | 0.03 | 0.0008 | -0.0008 | -0.001 | 0.006 | 0.01 | 0.03 | -0.002 | 0.001 | -0.05 | 0.007 | 0.03 | 0.004 | 0.04 | 0.05 | -0.04 | 0.06 | 0.007 | 0.001 |
| **Arnego** | 0.06 | - | 0.01 | 0.04 | 0.04 | 0.05 | 0.07 | 0.08 | 0.04 | 0.05 | 0.07 | 0.001 | 0.08 | 0.02 | 0.06 | 0.06 | 0.03 | 0.07 | 0.07 | 0.03 | 0.04 | 0.06 | 0.02 | 0.04 | 0.04 | 0.07 |
| **Beça Canedo** | 0.03 | 0.007 | - | -0.1 | 0.07 | 0.04 | 0.08 | 0.08 | 0.05 | -0.007 | 0.07 | 0.03 | 0.09 | 0.06 | 0.06 | 0.002 | 0.02 | 0.05 | 0.03 | 0.06 | -0.0007 | -0.12 | 0.02 | -0.06 | -0.02 | 0.05 |
| **Beça Gondiaes** | 0.008 | 0.007 | 0.89 | - | 0.06 | 0.06 | 0.1 | 0.1 | 0.07 | 0.03 | 0.08 | 0.03 | 0.1 | 0.04 | 0.08 | 0.04 | 0.04 | 0.07 | 0.06 | 0.06 | 0.03 | -0.06 | 0.04 | -0.04 | 0.01 | 0.09 |
| **Bibey** | 0.008 | 0.007 | 0.007 | 0.008 | - | 0.05 | 0.08 | 0.07 | 0.06 | 0.06 | 0.05 | 0.05 | 0.06 | 0.04 | 0.08 | 0.07 | 0.02 | 0.08 | 0.09 | 0.07 | 0.08 | 0.08 | 0.03 | 0.09 | 0.07 | 0.07 |
| **Eo** | 0.04 | 0.006 | 0.009 | 0.007 | 0.008 | - | 0.08 | 0.05 | 0.05 | 0.05 | 0.04 | -0.03 | 0.06 | -0.001 | 0.07 | 0.07 | -0.005 | 0.07 | 0.1 | -0.04 | 0.06 | 0.09 | 0.006 | 0.05 | 0.06 | 0.08 |
| **Esqueiro** | 0.008 | 0.008 | 0.008 | 0.008 | 0.007 | 0.008 | - | 0.09 | 0.05 | 0.09 | 0.08 | 0.03 | 0.08 | 0.04 | -0.06 | 0.1 | 0.06 | 0.1 | 0.12 | 0.07 | 0.11 | 0.12 | 0.04 | 0.1 | 0.1 | 0.12 |
| **Esva** | 0.007 | 0.007 | 0.007 | 0.008 | 0.008 | 0.008 | 0.007 | - | 0.07 | 0.09 | 0.04 | 0.002 | 0.05 | 0.02 | 0.09 | 0.11 | 0.03 | 0.12 | 0.13 | -0.03 | 0.12 | 0.11 | 0.04 | 0.1 | 0.09 | 0.1 |
| **Mandeo** | 0.01 | 0.009 | 0.007 | 0.008 | 0.007 | 0.008 | 0.007 | 0.008 | - | 0.08 | 0.07 | 0.03 | 0.06 | 0.04 | 0.04 | 0.08 | 0.03 | 0.08 | 0.09 | 0.04 | 0.08 | 0.1 | 0.02 | 0.09 | 0.08 | 0.1 |
| **Mente** | 0.009 | 0.007 | 0.008 | 0.009 | 0.007 | 0.007 | 0.006 | 0.007 | 0.009 | - | 0.09 | 0.03 | 0.1 | 0.04 | 0.07 | 0.04 | 0.04 | 0.07 | 0.08 | 0.07 | -0.07 | 0.03 | 0.04 | 0.003 | 0.008 | 0.11 |
| **Narcea 1** | 0.01 | 0.008 | 0.006 | 0.008 | 0.008 | 0.007 | 0.006 | 0.009 | 0.008 | 0.007 | - | 0.35 | -0.02 | -0.06 | 0.07 | 0.08 | 0.03 | 0.07 | 0.09 | 0.01 | 0.11 | 0.1 | 0.04 | 0.09 | 0.1 | 0.1 |
| **Narcea 2** | 0.03 | 0.009 | 0.03 | 0.007 | 0.007 | 0.049 | 0.009 | 0.008 | 0.008 | 0.008 | -0.5 | - | 0.002 | -0.02 | 0.02 | 0.03 | -0.03 | 0.05 | 0.06 | -0.03 | 0.06 | 0.06 | -0.02 | 0.04 | 0.04 | 0.03 |
| **Narcea 3** | 0.03 | 0.006 | 0.03 | 0.007 | 0.007 | 0.006 | 0.008 | 0.007 | 0.009 | 0.008 | 0.05 | 0.03 | - | 0.26 | 0.03 | 0.08 | 0.02 | 0.07 | 0.09 | 0.04 | 0.11 | 0.12 | 0.04 | 0.1 | 0.1 | 0.09 |
| **Narcea 4** | 0.03a | 0.007 | 0.03 | 0.007 | 0.008 | 0.01 | 0.008 | 0.008 | 0.008 | 0.007 | 0.61 | 0.03 | -0.05 | - | 0.03 | 0.03 | -0.03 | 0.04 | 0.06 | 0.01 | 0.06 | 0.06 | -0.01 | 0.06 | 0.04 | 0.05 |
| **Navia** | 0.02 | 0.008 | 0.03 | 0.009 | 0.008 | 0.01 | 0.31 | 0.007 | 0.007 | 0.009 | 0.007 | 0.03 | 0.08 | 0.04 | - | 0.1 | 0.04 | 0.09 | 0.11 | 0.05 | 0.09 | 0.1 | 0.03 | 0.09 | 0.09 | 0.11 |
| **Neiva** | 0.02 | 0.008 | 0.01 | 0.008 | 0.007 | 0.007 | 0.008 | 0.009 | 0.007 | 0.007 | 0.009 | 0.008 | 0.007 | 0.009 | 0.007 | - | 0.05 | 0.08 | 0.06 | 0.07 | 0.03 | 0.05 | 0.06 | 0.03 | 0.03 | 0.12 |
| **Ouro** | 0.05 | 0.008 | 0.03 | 0.008 | 0.01 | 0.02 | 0.008 | 0.008 | 0.007 | 0.007 | 0.008 | 0.08 | 0.03 | 0.15 | 0.03 | 0.009 | - | 0.05 | 0.07 | -0.02 | 0.05 | 0.07 | -0.03 | 0.04 | 0.05 | 0.07 |
| **Paiva Castro Daire** | 0.03 | 0.009 | 0.03 | 0.007 | 0.007 | 0.008 | 0.01 | 0.007 | 0.007 | 0.008 | 0.007 | 0.03 | 0.03 | 0.03 | 0.02 | 0.008 | 0.03 | - | -0.04 | 0.08 | 0.09 | 0.1 | 0.05 | 0.07 | 0.09 | 0.12 |
| **Paiva Várzea** | 0.008 | 0.006 | 0.008 | 0.009 | 0.008 | 0.006 | 0.007 | 0.008 | 0.007 | 0.008 | 0.007 | 0.009 | 0.007 | 0.007 | 0.008 | 0.008 | 0.008 | 0.008 | - | 0.1 | 0.07 | 0.07 | 0.07 | 0.06 | 0.06 | 0.13 |
| **Porcia** | 0.07 | 0.04 | 0.06 | 0.046 | 0.05 | 0.09 | 0.05 | 0.04 | 0.05 | 0.04 | 0.05 | 0.07 | 0.06 | 0.07 | 0.07 | 0.04 | 0.14 | 0.06 | 0.04 | - | 0.09 | 0.09 | -0.02 | 0.08 | 0.06 | 0.03 |
| **Rabaçal** | 0.01 | 0.007 | 0.006 | 0.009 | 0.008 | 0.008 | 0.01 | 0.009 | 0.007 | 0.49 | 0.008 | 0.008 | 0.008 | 0.007 | 0.007 | 0.007 | 0.007 | 0.007 | 0.008 | 0.05 | - | 0.02 | 0.04 | 0.002 | -0.0003 | 0.1 |
| **Tâmega** | 0.008 | 0.007 | 0.99 | 0.18 | 0.008 | 0.006 | 0.007 | 0.01 | 0.009 | 0.008 | 0.008 | 0.008 | 0.008 | 0.009 | 0.008 | 0.007 | 0.009 | 0.008 | 0.008 | 0.05 | 0.006 | - | 0.06 | -0.04 | 0.01 | 0.11 |
| **Tambre** | 0.12 | 0.01 | 0.007 | 0.008 | 0.007 | 0.008 | 0.007 | 0.009 | 0.009 | 0.01 | 0.008 | 0.06 | 0.008 | 0.04 | 0.007 | 0.009 | 0.007 | 0.008 | 0.006 | 0.05 | 0.008 | 0.008 | - | 0.04 | 0.06 | 0.06 |
| **Terva** | 0.009 | 0.007 | 0.01 | 0.008 | 0.007 | 0.008 | 0.009 | 0.007 | 0.007 | 0.007 | 0.008 | 0.008 | 0.009 | 0.007 | 0.008 | 0.009 | 0.007 | 0.009 | 0.007 | 0.05 | 0.01 | 0.008 | 0.008 | - | -0.01 | 0.09 |
| **Tuela** | 0.007 | 0.008 | 0.007 | 0.007 | 0.008 | 0.007 | 0.008 | 0.009 | 0.008 | 0.01 | 0.007 | 0.008 | 0.009 | 0.009 | 0.009 | 0.007 | 0.007 | 0.008 | 0.007 | 0.05 | 0.007 | 0.008 | 0.006 | 0.008 | - | 0.11 |
| **Ulla** | 0.04 | 0.007 | 0.006 | 0.007 | 0.007 | 0.007 | 0.009 | 0.007 | 0.008 | 0.009 | 0.009 | 0.008 | 0.009 | 0.008 | 0.008 | 0.007 | 0.008 | 0.007 | 0.008 | 0.05 | 0.007 | 0.008 | 0.008 | 0.008 | 0.009 | - |

**Table S4. AMOVA analysis. * significant at 0.05 p-level**

| **Source of variation** | **df** | **sum of squares** | **Variance components** | **Percentage of variation** |
| --- | --- | --- | --- | --- |
| Among hydrological sub-basins | 15 | 13319.602 | 38.78970 | 8.54* |
| Among locations within hydrological sub-basins | 10 | 3258.804 | -11.33090 | -2.47* |
| among locations | 212 | 91359.800 | 430.94245 | 94.01* |
| Total | 237 | 107938.206 | 458.40126 |  |

**Supplementary Figures**

**Figure S1- sNMF (A1: K=2, A2: K=3) and Admixture (B1: K=2, B2: K=3) analyses for 26 hydrological locations**

**
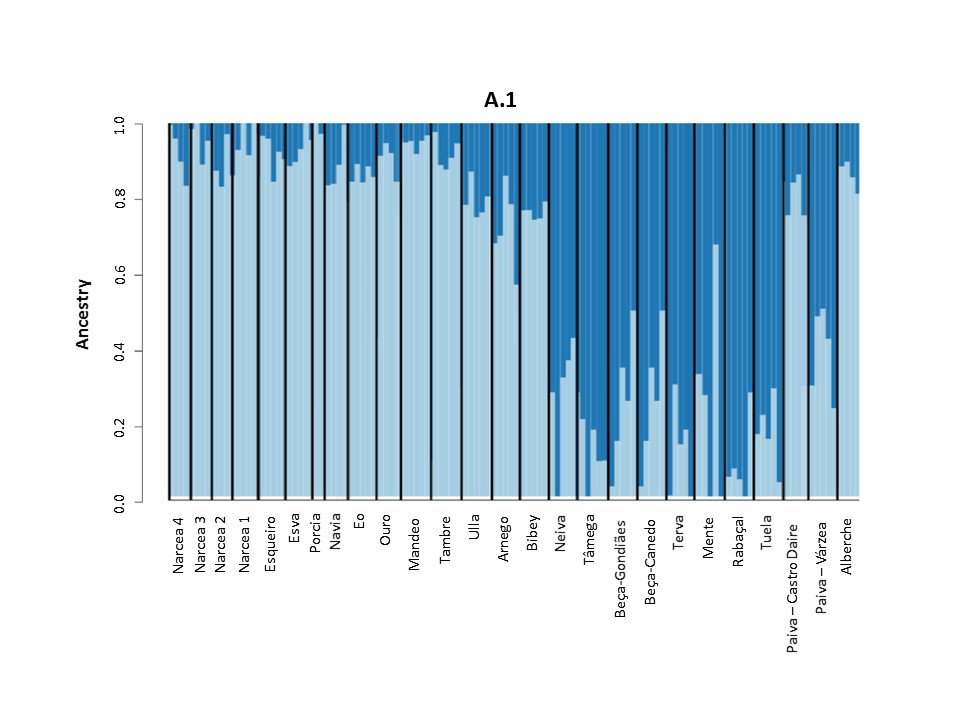
**

**
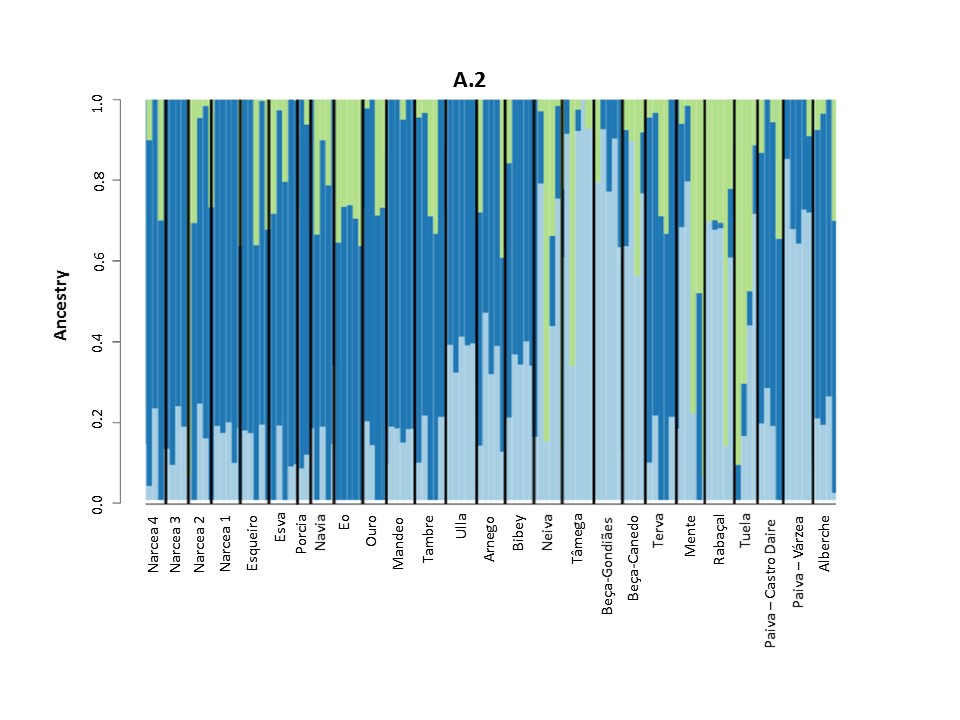
**

**
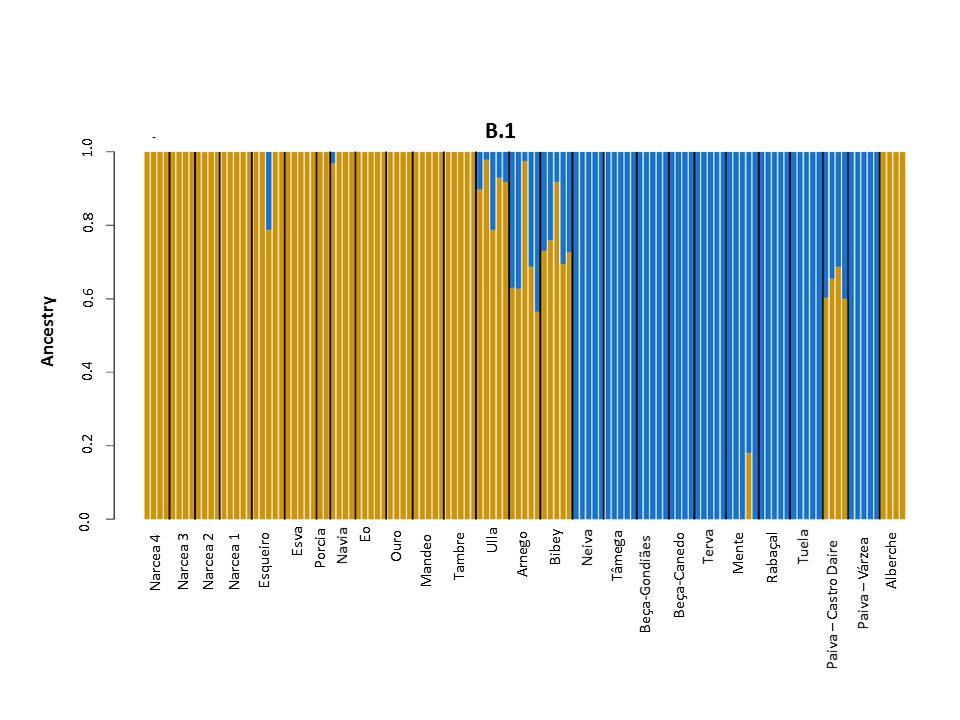

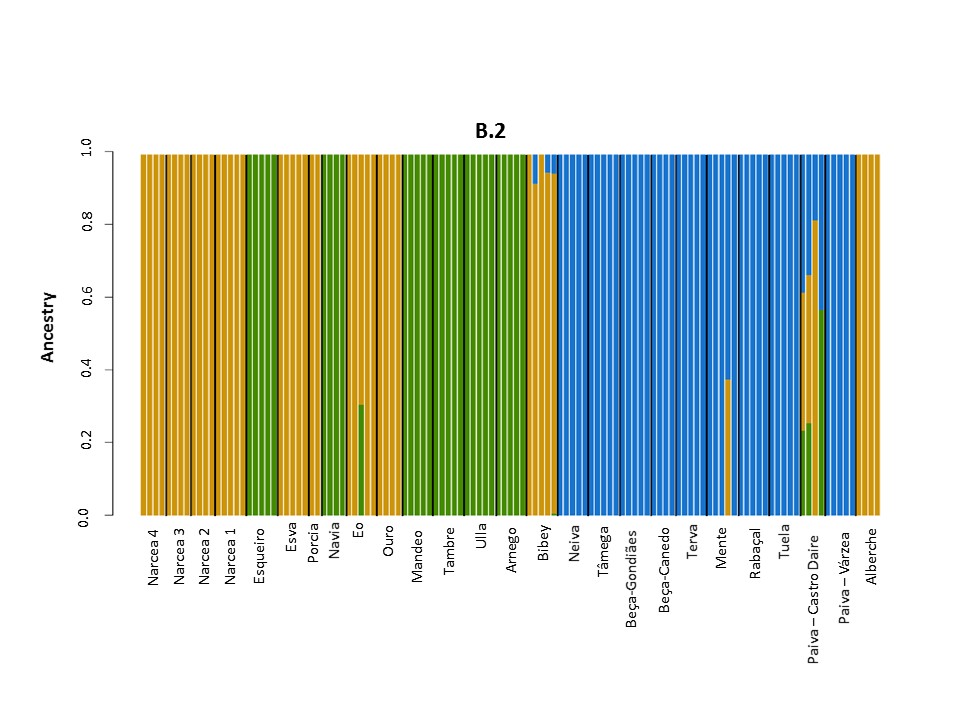
**

**Figure S2 – (A) Cross-entropy for each number of K ancestral populations inferred with sNMF. (B) Mean cross-validation error across 100 ADMIXTURE runs. For each value of K (1 to 16), the point represents the mean cross-validation error of the 100 ADMIXTURE runs performed.**

**
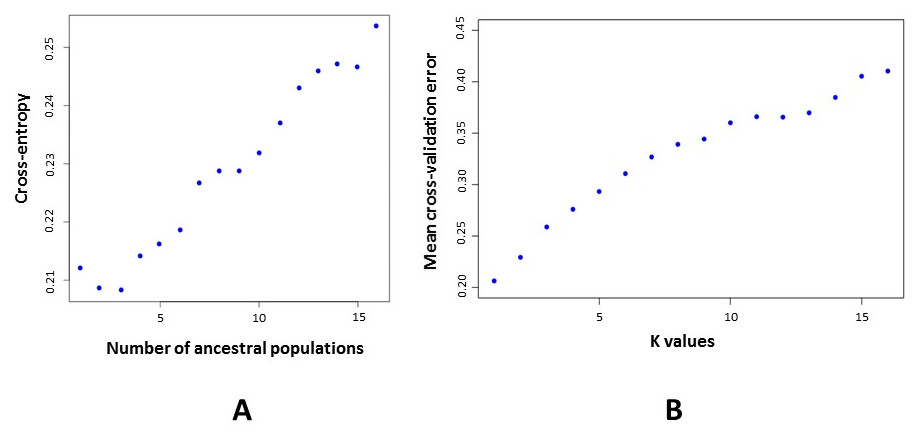
**
